# Histone Deacetylase Inhibitor Induced Radiation Sensitization Effects on Human Cancer Cells after Photon and Hadron Radiation Exposure

**DOI:** 10.3390/ijms19020496

**Published:** 2018-02-07

**Authors:** Ariungerel Gerelchuluun, Junko Maeda, Eri Manabe, Colleen A. Brents, Takeji Sakae, Akira Fujimori, David J. Chen, Koji Tsuboi, Takamitsu A. Kato

**Affiliations:** 1 Proton Medical Research Center, Faculty of Medicine, University of Tsukuba, Tsukuba, Ibaraki Prefecture 305-8575, Japan; ariukaa_t@yahoo.co.jp (A.G.); mana-e@hotmail.co.jp (E.M.); takejisakae@gmail.com (T.S.); 2 Department of Environmental & Radiological Health Sciences, Colorado State University, Fort Collins, CO 80523, USA; junkorv0507@yahoo.co.jp (J.M.); cbrents1@rams.colostate.edu (C.A.B.); 3 Department of Basic Medical Sciences for Radiation Damages, National Institute of Radiological Sciences, National Institutes for Quantum and Radiological Science and Technology, Chiba, Chiba Prefecture 263-8555, Japan; fujimori.akira@qst.go.jp; 4 Department of Radiation Oncology, University of Texas Southwestern Medical Center, Dallas, TX 75390, USA; david.chen@utsouthwestern.edu

**Keywords:** proton, carbon ions, SAHA

## Abstract

Suberoylanilide hydroxamic acid (SAHA) is a histone deacetylase inhibitor, which has been widely utilized throughout the cancer research field. SAHA-induced radiosensitization in normal human fibroblasts AG1522 and lung carcinoma cells A549 were evaluated with a combination of γ-rays, proton, and carbon ion exposure. Growth delay was observed in both cell lines during SAHA treatment; 2 μM SAHA treatment decreased clonogenicity and induced cell cycle block in G1 phase but 0.2 μM SAHA treatment did not show either of them. Low LET (Linear Energy Transfer) irradiated A549 cells showed radiosensitization effects on cell killing in cycling and G1 phase with 0.2 or 2 μM SAHA pretreatment. In contrast, minimal sensitization was observed in normal human cells after low and high LET radiation exposure. The potentially lethal damage repair was not affected by SAHA treatment. SAHA treatment reduced the rate of γ-H2AX foci disappearance and suppressed RAD51 and RPA (Replication Protein A) focus formation. Suppression of DNA double strand break repair by SAHA did not result in the differences of SAHA-induced radiosensitization between human cancer cells and normal cells. In conclusion, our results suggest SAHA treatment will sensitize cancer cells to low and high LET radiation with minimum effects to normal cells.

## 1. Introduction

Radiosensitizers can be beneficial for radiation therapy via increasing cell killing [1]. However, radiosensitizers are still under trial and testing. Therefore, it is important to search clinically approved anti-cancer drugs for novel radiosensitizing effects to avoid complications during drug trials. Second-generation histone deacetylase inhibitor (HDACi), suberovlanilide hydroxamic acid (SAHA; commercial name, vorinostat) is a USA Food and Drug Administration (FDA)-approved drug for cutaneous T cell lymphoma treatment [2,3]. In vitro and in vivo studies suggested that HDACi could be used to treat many types of cancers [4]. HDACi has portrayed a broad spectrum of epigenetic activities by the accumulation of acetylated histones and acetylated non-histone proteins. Several studies have shown HDACi sensitizes cancer stem-like cells to damaging agents [5,6,7]. Recent publications have reported not only anti-tumor effects but also radiosensitization effects of SAHA through DNA double strand break repair inhibition [8,9,10]. One pathway identified in these publications was the homologous recombination repair pathway [11,12,13,14], and another pathway was non-homologous end joining repair through Ku70 and 86 [15,16]. These DNA repair inhibitory effects by HDACi were combined with photon ionizing radiation and chemotherapeutic agents, and showed synergistic effects for cell killing. However, a combination of HDACi with particle radiation, including proton and carbon ion, are still limited [17,18,19,20].

As an anti-tumor drug, SAHA has selective cytotoxicity in transformed normal cells and cancer cells [7]. Cells with a mutation in p53 are sensitive to SAHA. In addition, HDACi may have a protective role in normal cells by suppressing the oncogenes, radiation-induced inflammatory cytokines, and stimulating the proliferation of stem cells [21,22]. Other factors such as extended phosphorylation of H2AX may be associated with this selective toxicity and radiosensitization [23,24]. Since cytotoxicity and radiation sensitivity often share the same mechanisms, SAHA’s radiosensitization effect may be also selective to cancer cells. One of the reasons why radiosensitizers are still in testing or trial is that drugs often sensitize both normal and cancer cells. For example, BrdU is a classical radiosensitizer for highly proliferative cells such as cancer cells [1]. Caffeine which is commonly available in various beverages can suppress cell cycle checkpoint and sensitize cells to radiation [25]. Neither of the latter chemicals portray clinical relevance, due to their high toxicity and non-selective sensitization effects. Therefore, the potentially selective radiosensitization effect of SAHA is useful in a clinical setting. One of the advantages of hadron radiation therapy is an excellent dose distribution within the target tumor volume. Late effects of proton and carbon ion therapy has been shown to be superior to that of intensity modulated radiation therapy [26]. In radiotherapy, reducing the therapy dose is of particular importance. Therefore, SAHA might be a suitable combined modality for hadron radiation therapy.

In this paper, we hypothesize that the anti-tumor drug SAHA selectively sensitizes cancer cells to DNA damage from both photon radiation and hadron radiation. We have investigated the selective sensitization with SAHA treatment in cancer cells versus normal cells. Furthermore, we explored the DNA damage response and repair to understand mechanisms of selective radiosensitization by SAHA.

## 2. Results

### 2.1. Cellular Toxicity and Cell Cycle Distribution from SAHA Treatment

**Figure 1:**
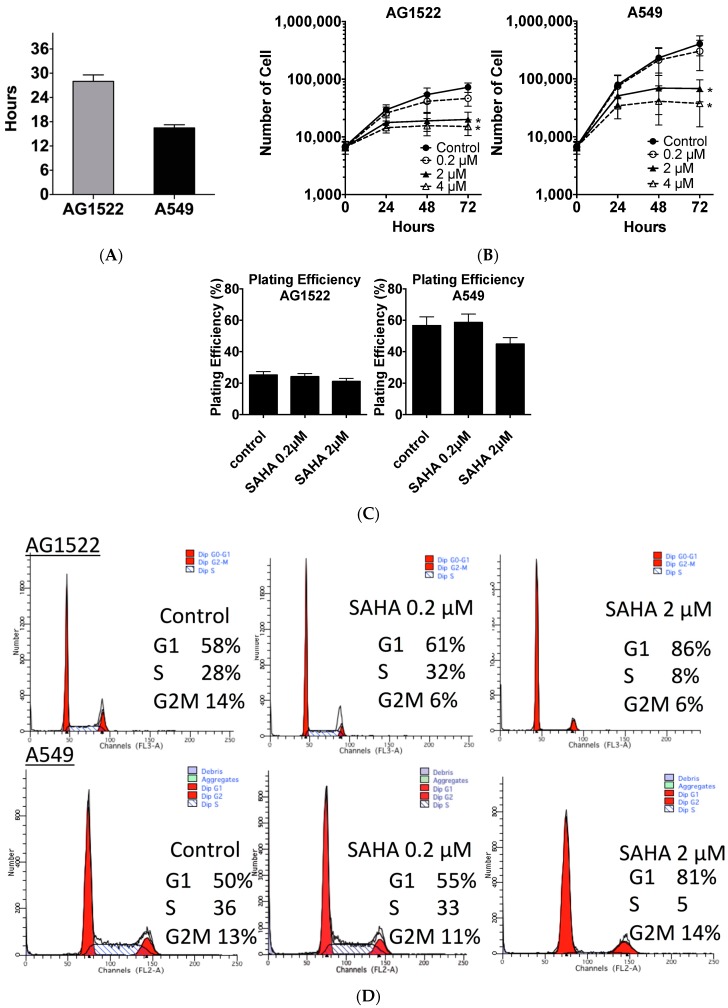

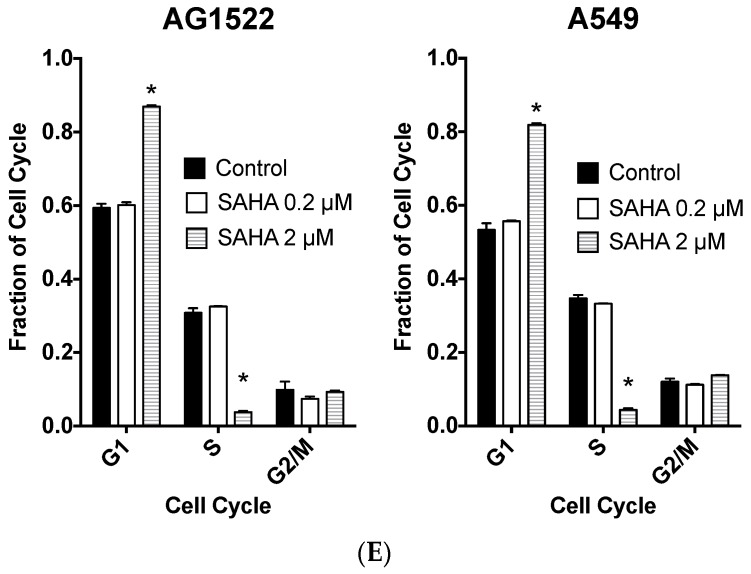
Suberoylanilide hydroxamic acid (SAHA)’s effects to human normal fibroblast and cancer cell culture conditions. (**A**) Cellular doubling times of AG1522 and A549; (**B**) Cellular toxicity evaluated by cell growth for three days in the presence SAHA; (**C**) Cellular toxicity evaluated by clonogenic ability as plating efficiency; (**D**) Flow cytometry profiles after SAHA treatment for 24 h; and (**E**) Cell cycle distribution after 24 h SAHA treatment. Error bars indicate standard error of the means. * marks mean statistically significant differences compared to control (*p* < 0.05). All experiments were carried out at least three times independently.

Cell doubling time of AG1522 normal human fibroblast cells was approximately 28 h. That of A549 human lung carcinoma was approximately 16 h (Figure 1A). Three-day SAHA continuous treatment reduced cellular growth rate in both normal and cancer cell lines (Figure 1B). Log growing cells were exposed to different concentrations of SAHA (0.2–4 μM). Dose dependent cell growth inhibition was observed for both cell lines in the tested range, 0.2 to 4 μM (Figure 1B). Also, 2 μM and 4 μM SAHA treatment showed significant growth inhibition at 3 days compared to control (*p* < 0.05).

In order to determine whether SAHA-reduced growth was associated with cell death, a clonogenic survival test was carried out after 24 h SAHA treatment. Plating efficiency without SAHA treatment of AG1522 was 25% and A549 was 55% in our experimental conditions. Clonogenic survival showed a slight reduction of clonogenicity with the high concentration (2 μM) treated AG1522 was 20% and A549 was 45% and no toxic effects were seen at a low concentration (0.2 μM) treatment (Figure 1C). However, no statistical significance for the different conditions was observed in both AG1522 and A549 cells (ANOVA (Analysis of Variance) *p* = 0.115 and *p* = 0.345, respectively).

Cell cycle distribution after 24 h 2 μM SAHA treatment presented cell cycle arrest for both normal cells and cancer cells in G1 phase (Figure 1D,E). G1 phase population increased from 50–60% to more than 80%, with statistical significance (*p* < 0.05). S phase population was decreased from 30% to 5%, with statistical significance (*p* < 0.05). G2/M phase population remained the same. Therefore, the slower cell division by SAHA treatment may arise from temporary cell division arrest, such as activation of the cell cycle checkpoints but not permanent senescence.

### 2.2. Radiosensitization of Exponentially Growing Cancer Cells Exposed to γ-Rays, Protons and Clinical Grade Carbon Ions 

**Figure 2:**
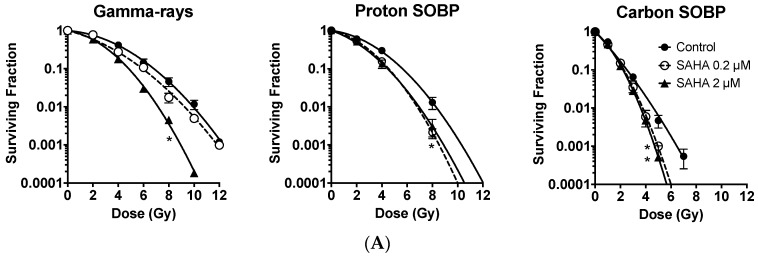

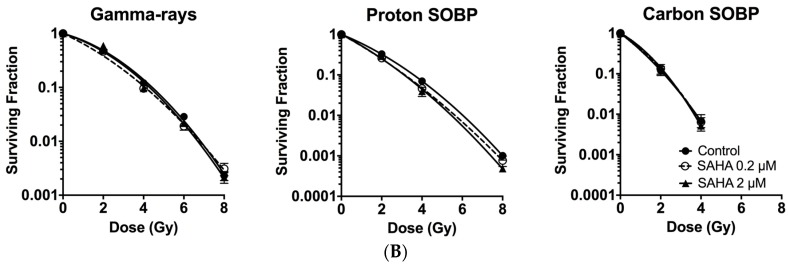
SAHA induced radiosensitization for exponentially growing normal and cancer cells. (**A**) SAHA induced radiosensitization in exponentially growing A549 cancer cells; (**B**) No radiosensitization effect by SAHA treatment for exponentially growing AG1522 normal cells. Error bars indicate standard error of the means. * marks mean statistically significant differences compared to control (*p* < 0.05). All experiments were carried out at least three times independently.

Two concentrations of SAHA (0.2 and 2 μM) were used for radiosensitization tests for the exponentially growing A549 cells. It was found that 2 μM of SAHA pretreatment resulted in statistically significant radiosensitization for γ-rays, proton SOBP (Spread out Bragg Peak), and carbon ion SOBP (*p* < 0.05). In contrast, 0.2 μM of SAHA pretreatment induced statistically significant radiosensitization with proton SOBP and carbon ion SOBP (*p* < 0.05) but not γ-rays (Figure 2A). Although low concentrations of SAHA did not show any cell cycle differences from control (Figure 1D), it showed similar sensitization effects to protons and carbon ion exposure for A549 lung cancer cell line when compared to high concentrations of SAHA.

D_10_ values and Sensitization Enhancement Ratio (SER) values of each condition were summarized in Table 1. Relative Biological Effectiveness (RBE) values for A549 obtained from the D_10_ values for each condition showed RBE 1.24 for proton and 2.59 for carbon SOBP irradiation without SAHA treatment. SER values 1.18 and 1.43 for γ-rays; 1.27 and 1.31 for protons; and 1.15 and 1.18 for carbon-ion (low and high concentrations, respectively) (Table 1). High concentrations showed slightly stronger sensitization and slightly higher SER values. SAHA-induced radiosensitization was LET (Linear Energy Transfer) dependent. SAHA sensitized low LET radiation (γ-rays and protons) to a greater extent than high LET carbon ion radiation when sensitization effects were compared with SER. These results suggest that SAHA is expected to have positive synergistic effects with proton radiation, rather than high LET carbon ion exposure for cancer cell killing. On the other hand, at high doses (5 Gy), low or high concentration of SAHA treatment successfully sensitized cells to carbon ion irradiation (*p* < 0.05).

### 2.3. Minimum Radiosensitization Effect of SAHA for Exponentially Growing Normal Cells

In order to compare radiosensitization effects with cancer cells, normal human fibroblast AG1522 was used initially in exponentially log growth conditions. SAHA showed minimum radiosensitization effects to this normal cell line when it was combined with γ-rays, protons, or carbon-ions (Figure 2B).

AG1522’s RBE values without SAHA treatment were 1.25 for protons and 2.18 for carbon ions. SER values were 1.03 and 1.08 for γ-rays; 1.13 and 1.16 for protons; and 1.05 and 0.95 for carbon-ion (low and high concentrations, respectively) (Table 1). SER values for normal cells were smaller than cancer cells. Therefore, in log phase cells, SAHA’s radiosensitization is specific and stronger in cancer cells.

### 2.4. G0/G1 Phase Cells and Potentially Lethal Damage Repair with Immediate or Delayed Subculture

**Figure 3:**
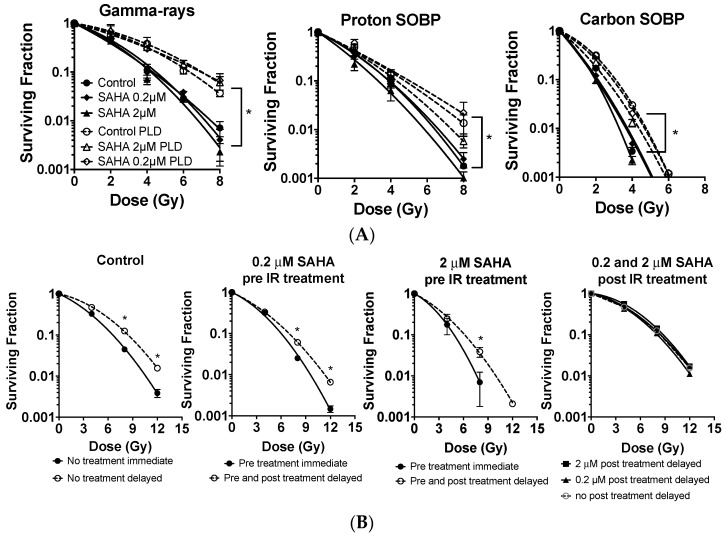

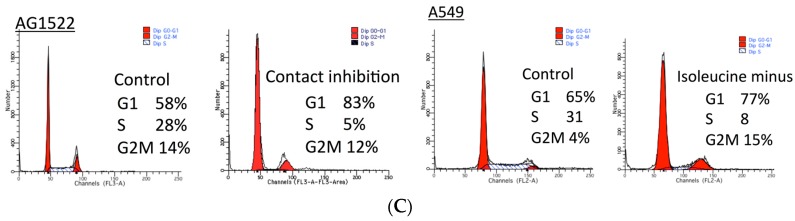
SAHA induced radiosensitization for synchronized cell population and potentially lethal damage recovery. (**A**) The effect of SAHA treatment for potentially lethal damage repair in contacted inhibited G0/G1 phase AG1522 cells after γ-rays, proton, and carbon-ion exposure; (**B**) The effect of SAHA treatment for potentially lethal damage repair in G1 phase synchronized A549 cancer cells with γ-rays, proton, and carbon-ion exposure; (**C**) DNA profiles for control and after synchronization. Error bars indicate standard error of the means. * marks mean statistically significant differences between immediate and delayed subculture (*p* < 0.05). All experiments were carried out at least three times independently.

In order to eliminate any cell cycle distribution effects to evaluate SAHA’s radionsensitization, a further study was carried out in the synchronized cell population (Figure 3) and summarized to Table 2. After more than 80% of normal cells were synchronized in G0/G1 phase by contact inhibition, SAHA treatment was carried out 24 h prior to the irradiation. SAHA was kept in media for 24 h post irradiation recovery period. When SAHA was absent, normal cells showed a clear increase of cell survival with 24 h delayed subculture, compared to immediate subculture as potentially lethal damage repair. The D_10_ values were increased from 4.31 Gy to 6.35 Gy by 24 h delayed subculture with γ-rays (Table 2). Therefore, normal cells showed potentially lethal damage repair. Increased D_10_ values with 24 h delayed subculture was observed greater in γ-rays, intermediate with protons, and the lowest with carbon ions. SAHA pretreatment did not affect cell survival for normal human fibroblast cells for immediate subculture or delayed subculture (Figure 3A). Therefore, SAHA treatment did not sensitize G0/G1 synchronized normal human fibroblasts and did not affect potentially lethal damage repair.

In order to investigate SAHA’s effect for further potentially lethal damage repair, SAHA treatment was carried out with more than 75% of G1 synchronized A549 cells. Initially, potentially lethal damage repair was confirmed in G1 phase A549 cells after γ-ray irradiation (Figure 3B) with significant increase of cell survival with delayed subculture compared to immediate subculture (*p* < 0.05). SAHA pretreatment sensitized cells, both immediate subcultured and delayed subcultured cells to radiation in the presence of SAHA. Also, 24 h delayed subculture showed significant increased cell survival compared to immediate subculture (Figure 3B) (*p* < 0.05). In order to clarify whether SAHA sensitized A549 or affected potentially lethal damage repair, SAHA treatment was carried out by adding SAHA immediately after irradiation and subculture 24 h later. Post irradiaiton treatment of SAHA did not affect cell survival and did not inhibit potentially lethal damage repair. Therefore, SAHA did not show any inhibition of potentially lethal damage repair in normal and cancer cells.

### 2.5. DSB (Double Starand Break) Repair

**Figure 4:**
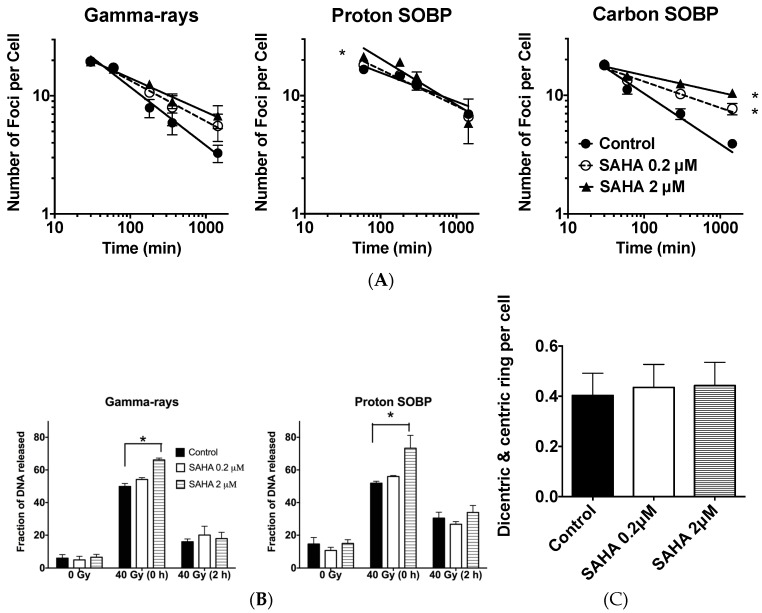
SAHA induced radiosensitization in DNA damage and repair. (**A**) γ-H2AX foci kinetics in AG1522 cell lines after 1 Gy of different radiation qualities; (**B**) Constant field gel electrophoresis analysis in A549 after 40 Gy of radiation; (**C**) Chromosome aberrations after 4 Gy of γ-ray irradiation to AG1522. Error bars indicate standard error of the means. All experiments were carried out at least three times independently. * marks indicate statistically significant differences (*p* < 0.05).

As a molecular indicator for DNA double strand break and repair, radiation induced γ-H2AX foci formation and disappearance were analyzed with SAHA treatment. An initial number of foci at 30 or 60 min after radiation exposure did not change with or without SAHA treatment with γ-ray exposure and carbon ion exposure for AG1522 cells. However, with proton exposure, 2 μM SAHA treatment significantly increased foci number (Figure 4A). Foci number decreased with time after radiation exposure. SAHA treatment reduced the rate of foci disappearance. Then, 24 h after radiation exposure, approximately two times more foci were observed with high concentration of SAHA treatment following treatment with γ-rays without statistical significance and three times for carbon-ion exposure with statistical significance (*p* < 0.05) (Figure 4A) but no differences for proton exposure.

As a direct indicator for DNA double strand break repair, low voltage constant field gel electrophoresis was carried out to confirm SAHA’s effect in DNA double strand break formation and repair with A549 cells. SAHA treatment slightly increased initial DNA double strand break formation after γ-rays and proton exposure, which was evaluated as migrated DNA from well with statistical significance (*p* < 0.05) (Figure 4B). However, differences in residual DNA double strand breaks between each sample were not detected at 2 h after either γ-rays or proton irradiation of 40 Gy in A549 cells (Figure 4B). 

### 2.6. Chromosomal Aberrations

SAHA treatment did not affect radiation induced chromosomal aberration formation (dicentric and ring chromosome formation) in normal human fibroblasts after 4 Gy of γ-ray exposure (Figure 4C). Human normal fibroblast cells were not sensitized for cell killing and chromosomal aberration formation with SAHA treatment combined with γ-rays.

### 2.7. Rad51 and RPA (Replication Protein A) Focus Formation

**Figure 5:**
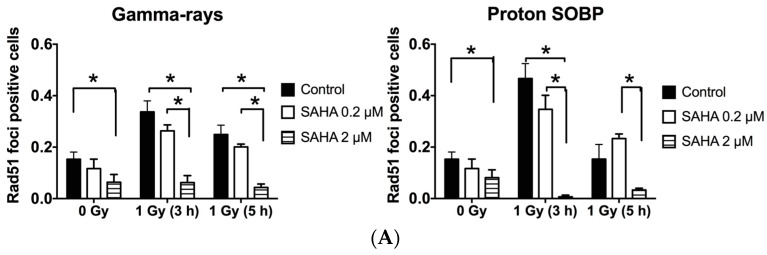

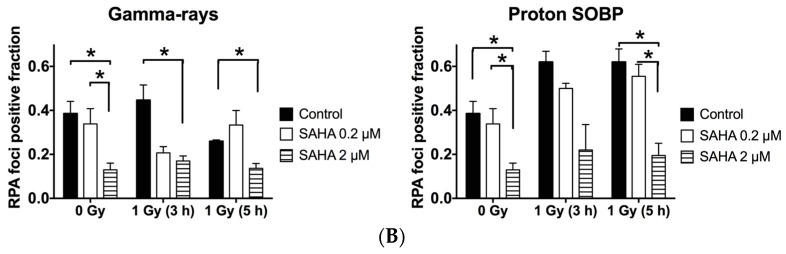
SAHA induced radiosensitization in homologous recombination repair. (**A**) Rad51 foci positive fraction in A549 cells; (**B**) RPA (Replication Protein A) foci positive fraction in A549 cells. Error bars indicate standard error of the means. All experiments were carried out at least three times independently. * marks indicate statistically significant differences (*p* < 0.05).

SAHA’s effect on homologous recombination repair was analyzed by Rad51 and RPA foci formation at 3 and 5 h after 1 Gy of γ-ray or proton beam radiation in cancer cell line. SAHA treatment suppressed Rad51 and RPA foci formation in control and γ-ray and proton beams irradiated samples, and it was significant for 2 μM of SAHA (Figure 5A,B). 

## 3. Discussion

Although multiple radiosensitizers were investigated and proposed for clinical uses, there are no clinically relevant radiosensitizers yet available. One of the major problems is non-selective radiosensitization between cancer cells and normal cells. In order to overcome this issue, several strategies including targeting drug delivery systems have been investigated [27]. Even SAHA has been investigated for targeted drug delivery system to improve efficacy [28,29,30]. A potential solution is to search for drugs that can selectively sensitize cancer cells to ionizing radiation, while neglecting normal cells. Significantly, anti-tumor drugs with the selective radio-sensitizing properties are most desirable because of its double synergistic effects; anti-tumor cell killing and tumor-selective radio-sensitization. SAHA is an anti-tumor drug and it is known that the cytotoxic effects are limited to transformed and cancerous cells [31]. Many reports showed SAHA induced radio-sensitization in cancer cells with photon and hadron radiation [17,18,19,20,32]. A prior study showed SAHA’s selective radiosensitization to cancer cells (sarcoma, glioblastoma, squamous cell carcinoma) with photon irradiation [23]. Our report expands this and is the first investigation to include a normal human fibroblast cell line and a human lung cancer cell line with photon, proton and carbon ion radiation together for SAHA’s selective radiosensitization (Figure 2 and Figure 3).

Hadron radiation more efficiently delivers dose in tumor volume when compared to photon radiation. For current clinically available hadron radiation, proton radiation surpasses carbon ion radiation for dose distribution [33]. The majority of carbon ions are fragmented by the collision with targets. As a result, the distal end of carbon ion irradiation has more dose deposition than proton irradiation [34]. This dose deposition at the distal end is important for the cancer treatment regarding evaluating patient’s secondary cancer risk and irreversible late effects such as radiation-induced lung fibrosis [33,35].

SAHA’s cancer specific radiosensitization has unclear mechanisms. A series of publications have shown that suppression of homologous recombination repair by HDACi treatment, including SAHA and homologous recombination associated RAD50 and MRE11 suppression, were observed in cancer cells only [10,11,14,32]. A recent study observed SAHA and cisplatin treated cancer cells showed hypersensitivity to radiation [32]. Both low and high concentrations of SAHA treatment successfully sensitized exponentially growing cancer cells (Figure 2A). S phase cells were significantly decreased and G1 arrest was seen in both cell lines after treatment with 2 μM SAHA, but not with low concentration 0.2 μM SAHA (Figure 1D,E). This may imply that low and high concentrations of SAHA’s cell sensitizing mechanism may be different from simple cell cycle distribution effects, such as accumulation of cell population into radiosensitive G1 phase [36,37]. It is known that HDACi increases p53 acetylation and activates cell cycle checkpoints by p21, resulting in cell arrest in G1 or G2/M [38]. SAHA induced arrest at G1 phase in AG1522 and A549, which have a p53 wild type status, might be the result of proper p53 function. Although we have also observed the reduction of Rad51 and RPA foci (Figure 5A,B), suppression of homologous recombination repair cannot explain the mechanism of neither cancer specific radiosensitization nor radiosensitization of G1 phase synchronized A549 cells.

Other reports showed suppression of non-homologous end joining repair-associated proteins including Ku86, Ku70 and DNA-PKcs with HDACi [15,39,40]. HDACi induced acetylation of Ku70 decreases its binding activity to DNA, resulting in impaired double strand break repair [16]. Reduction of non-homologous end joining repair should increase radiosensitivity in not only G1 phase, but also all cell cycle phases. Often, suppression of non-homologous end joining repair leads to suppression of potentially lethal damage repair [41]. We observed sensitization in G1 phase cancer cells with SAHA treatment but potentially lethal damage repair was intact with SAHA treatment (Figure 3). Therefore, the major mechanisms of radio-sensitization were not fully explained by simple DNA repair protein inhibitory model, such as suppression of Ku86 or Rad51. Furthermore, SAHA treatment extends the longevity of phosphorylation of H2AX signal [4]. Strikingly, we were able to observe this in normal human fibroblasts (Figure 4A), which were minimally sensitized by SAHA. Because we did not observe clear DNA double strand break repair reduction in constant field gel electrophoresis (Figure 4B), the ultimate mechanisms of SAHA-induced radiosensitization may be different from DNA double strand break repair.

HDACi is an epigenetic drug and has a wide range of action. Morphological changes of tumor cells, including chromatin texture, are one of the important criteria for cancer diagnosis. For successful repair of double strand break, chromatin needs to be fully decondensed for access of repair proteins to the site of DNA damage and the chromatin needs to be fully recondensed once the repair is completed, which is an essential process for accurate DNA repair [42]. The difference of chromatin structure in cancer cells may increase the accessibility of HDACi to the histone and may interfere restoration of chromatin once the repair is completed [43,44,45].

## 4. Materials and Methods

### 4.1. Cell Culture

A549, a lung small cell carcinoma and AG1522, a normal human fibroblast cell line, were kindly supplied from Dr. Joel Bedford at Colorado State University (Fort Collins, CO, USA). Cells were cultured in MEM-alpha (Gibco, Indianapolis, IN, USA) supplemented with 10% heat inactivated fetal bovine serum (Sigma, St Louis, MO, USA) for cancer cells and 15% for normal cells and 1% penicillin, streptomycin and Fungizone solution (anti-anti; Invitrogen Life Technologies, Carlsbad, CA, USA), and they were maintained at 37 °C in a humidified atmosphere of 5% CO_2_ in air. SAHA was purchased from Sigma. Then, 200 μM stock solution was prepared in dimethylsulfoxide (DMSO) and diluted to 0.2 μM and 2 μM of working solution in MEM-alpha. SAHA treatment was carried out for 24 h.

### 4.2. Cell Cycle Synchronization

For normal human fibroblasts, cells were synchronized in G0/G1 phase by contact inhibition. After cells reached into confluency, we changed media and waited for 2 days before experiment to ensure contact inhibition. For cancer cells, which cannot be synchronized by contact inhibition, cell culture medium was changed into isoleucine depleted MEM-media (Gibco) supplemented with 5% three times dialyzed serum and 1% penicillin, streptomycin and Fungizone solution as previously described [46,47]. Cells were incubated in this media for 24 h to synchronize them into G1 phase. Cell cycle synchronization was confirmed with FACSCalibur Flow Cytometer (BD Biosciences, Franklin Lakes, NJ, USA).

### 4.3. Irradiation

Cells were irradiated with either γ radiation, spread out Bragg peak (SOBP) of protons, or carbon-ions at room temperature. For γ-ray irradiation, a J. L. Shepherd Model Mark I-68 nominal 6000 Ci Cs-137 sealed source (J. L. Shepherd, Carlsbad, CA, USA) at the Colorado State University was used with a dose rate of 2.5 Gy per min.

For proton beam irradiation, 200 MeV proton beams were generated by the synchrotron at the Proton Medical Research Center (PMRC) of University of Tsukuba (Ibaraki, Japan). Proton dosimetry was measured as previously described [48,49]. The estimated energy spread and dose-average LET values at the middle of the 6-cm SOBP were 0–60 MeV and 2.2 keV/μm, respectively [49,50]. We classified middle of SOBP protons as low LET radiation. Proton beam irradiations were performed at the middle of the 6 cm SOBP with a dose rate of approximately 3 Gy/min.

Carbon-ion experiments were carried out using the heavy ion medical accelerator (HIMAC) at National Institute of Radiological Sciences (Chiba, Japan). Carbon ions were accelerated at 290 MeV/n of initial energy and spread out with a ridge filter for 6 cm width of SOBP. The monolayer culture in flasks was irradiated at the center within the SOBP at a distance of 119 mm from the entrance. The dose averaged LET of the carbon ions at the middle of 6 cm SOBP is about 50 keV/µm at the distance of 119 mm from the entrance with a dose rate of 1 Gy/min [51,52]. We classified middle of SOBP carbon ions as high LET radiation.

### 4.4. Cellular Toxicity and Cell Cycle Analysis

Firstly, 5000 cells were plated onto 6 well plates and cell number was counted by Coulter Counter Z1 (Beckman Coulter, Indianapolis, IN, USA) every 24 h for 5 days. Cell doubling time was calculated by using GraphPad Prism 6 with exponential growing equation from exponentially growing cell stage. Cellular toxicity was determined by growth inhibition in the presence of SAHA. Then, 7000 cells were exposed to 0, 0.2, 2, or 4 μM of SAHA for 3 days. Cell numbers of each condition were counted by Coulter Counter Z1.

For the clonogenicity test, after 24 h treatment of SAHA, cells were trypsinized to obtain single cell suspension. Then, 300–3000 cells were plated onto dishes to form colonies for 7–14 days. Dishes were fixed with ethanol and stained with 0.1% crystal violet. Macroscopic colonies containing more than 50 cells were marked as a survivor [53]. Plating efficiency (%) was calculated by observed colony number divided by plated single cell number.

Cell cycle analysis was carried out as previously described [54]. Briefly, after cells were exposed to SAHA for 24 h, cells were fixed in 70% ethanol and DNA was stained with Propidium iodide and followed by RNase treatment. Flow cytometry analysis was carried out with FACSCalibur and cell cycle distribution was determined with MODFIT LT version 3 software (Verity Software, Topsham, ME, USA) analysis.

### 4.5. Cell Survival Curves and RBE Analysis

After irradiation, cells were trypsinized and plated to form approximately 30–300 colonies. Cell survival curves were drawn from cell survival fraction by Graphpad Prism 6 (GraphPad, La Jolla, CA, USA) with linear quadratic regression model. D_10_ values (radiation dose to achieve 10% cell survival) were obtained. Relative biological effectiveness (RBE) and sensitization enhancement ratio (SER) values were calculated from D_10_ values. RBE values were calculated from the required γ-ray dose to kill 90% cells (D_10, γ_) divided by a testing radiation dose to kill 90% of cells (D_10, testing radiation_). RBE values more than 1 mean the testing radiation efficiently kills cells and has higher biological effectiveness compared to γ-rays. SER values were calculated from the dose required to kill 90% cells without sensitizer (D_10, control_) divided by a dose to kill 90% cells with testing sensitizer (D_10, sensitizer_). SER values higher than 1 mean testing sensitizer synergistically kills cells with radiation.

### 4.6. Potentially Lethal Damage Repair Analysis with Immediate or Delayed Subculture

**Figure 6:**
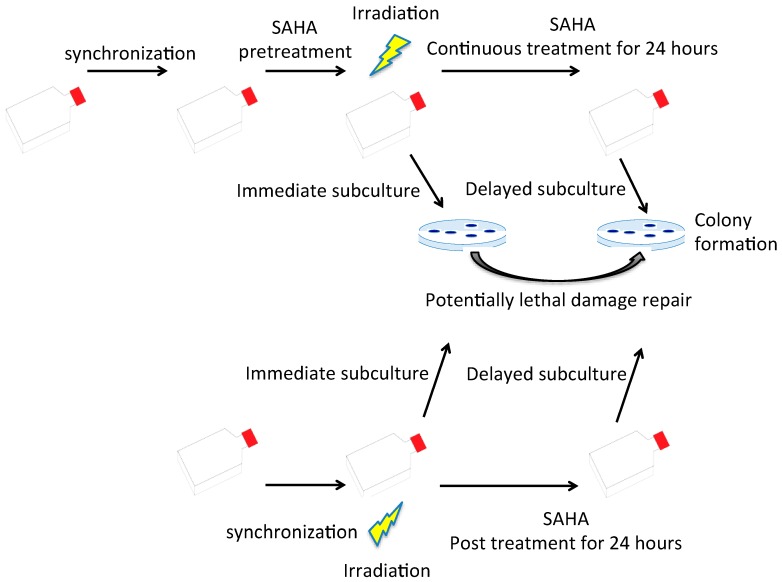
Potentially lethal damage repair analysis with immediate and delayed subculture. Synchronized cells were irradiated with or without SAHA treatment. After irradiation, cells were immediately subcultured for colony formation or delayed subculture after 24 h incubation in the presence of SAHA.

Potentially lethal damage repair is the recovery of radiation damage in the resting phase [55,56]. In order to determine whether SAHA in the media affects potentially lethal damage repair, G0/G1 synchronized cells were cultured with SAHA for 24 h before irradiation. After irradiation, they were immediately subcultured for colony formation to determine radiosensitivity without potentially lethal damage repair. On the other hand, after irradiation, cells were kept in the same media with SAHA for additional 24 h before trypsinization for colony formation (delayed subculture) to observe increased survival with potentially lethal damage repair. For the last group, 24 h SAHA treatment was started just after irradiation by adding SAHA to cell culture media and radiosensitivity was determined by colony formation assay after potentially lethal damage repair. Experimental procedures were summarized in Figure 6. Potentially lethal damage repair was analyzed by increased survival with greater D_10_ values obtained by cell survival curves.

### 4.7. Immunostaining and DNA Damage Response Analysis

γ-H2AX foci were analyzed for the contact inhibited G0/G1 AG1522 cells because S phase cells have a high background number of foci and γ-H2AX analysis is affected by cell cycle distribution. Rad51 and RPA foci were analyzed for the exponentially growing A549 cells. Cells were fixed in 4% paraformaldehyde for 15 min and treated for 5 min in 0.2% Triton X-100 (Sigma, St Louis, MO, USA) in PBS at room temperature, and blocked overnight at 4 °C in 10% goat serum solution. Immunostaining was carried out with mouse monoclonal primary antibody against γ-H2AX (Millipore, Billerica, MA, USA), Rad51 and RPA (Abram, Cambridge, MA, USA) and Alexa Fluor488-conjugated goat anti-mouse (Invitrogen Life Technologies). Cells were mounted with ProLong Gold Antifade Reagent with DAPI (Invitrogen Life Technologies). Then, 0.7 to 1.0-micron thick z-stack fluorescence images were captured using a Zeiss Axioskop motorized z-stage fluorescent microscope (Zeiss, Jena, Germany) equipped with CoolSNAP HQ2 (Photometrics, Tucson, AZ, USA) or Biozero BZ-8000 (Keyence, Tokyo, Japan). Extended focus images were obtained by Metamorph software (Molecular Devices, Sunnyvale, CA, USA). Three independent experiments were performed. At least 50 cells were analyzed for each experiment. Manual and Foci counter software-based counting was performed for foci analysis (http://focicounter.sourceforge.net/index.html).

Foci analysis shows the presence of repair proteins and phosphorylated form of histone proteins and indirect measurement of DNA damage and repair. In order to quantify actual DNA double strand break and repair, DNA double strand break was measured by the low voltage constant field gel electrophoresis method. This assay requires much higher doses compared to γ-H2AX foci assay. First, 250,000 cells were embedded in 0.5% low melting point agarose. After lysis with proteinase K and RNase treatment, electrophoresis was carried out in 0.5x TBE buffer in 0.6% agarose gel at room temperature in a constant field of 0.6 V/cm for 40 h. Gels were stained with Ethidium Bromide and the intensities of DNA which were retained in the plug and released into the lane were measured by Bio-rad ChemiDoc imager with Image Lab Software (Bio-Rad, Hercules, CA, USA). The fraction of DNA released was calculated from the amount of DNA in the plug and lane.

### 4.8. Chromosome Aberration Analysis

Contact inhibited G0/G1 phase AG1522 cells were pretreated with SAHA for 24 h before γ-ray irradiation. Immediately after irradiation, cells were trypsinized and replated. The first post irradiation metaphase cells were harvested with 0.1 μg/mL Colcemid (Invitrogen Life Technologies) treatment and prepared as a standard method [57]. Samples were treated in 75 mM KCl solution for 20 min at 37 °C and fixed in 3:1 (methanol: acetic acid) Carnoy fixation solution three times. Metaphase spreads were stained with 5% Giemsa solution, and the chromosome aberrations (dicentric chromosomes and ring chromosomes) were analyzed. A minimum of 100 metaphase cells was analyzed for each data point for at least two independent experiments.

### 4.9. Statistics

All experiments were carried out at least three times and error bars indicate standard error of the means. Data was analyzed using Prism 6 software for *t*-test and one-way ANOVA analysis. *p*-Values < 0.05 were categorized as significant differences.

## 5. Conclusions

Even though the molecular mechanisms of radiosensitization in cancer cells remain unclear, SAHA successfully sensitizes cells to radiation, even with high LET radiation. Importantly, we proved that SAHA selectively sensitized cancer cells to photon and hadron radiation. SAHA treatment did not affect potentially lethal damage repair. Potentially lethal damage repair is more important for low LET radiation, such as γ-rays and proton. This may be due to spatially distant DSBs, unable to interact or form lesions. On the other hand, high LET increases clusters of DSBs that contain the ability to form irreparable lesion. Based on the radiation dose control, enhancement ratio, and potential lower side effects for normal cells, SAHA treatment may be the most effective when combined with protons. Although our results show promising evidence, future studies utilizing other cell lines will be important to prove our hypothesis and strengthen the evidence that SAHA treatment can selectively kill cancer cells when combined with radiation.

## Figures and Tables

**Table 1:** SAHA-induced radiosensitization in A549 and AG1522 cells in different qualities of radiation. D_10_, Sensitization Enhancement Ratio (SER), and Relative Biological Effectiveness (RBE) values are mean ± standard error of the means.

Irradiation Condition	A549			AG1522		
	D10 (Gy)	SER	RBE	D10 (Gy)	SER	RBE
γ-rays Control	6.52 ± 0.33	NA	NA	4.42 ± 0.13	NA	NA
SAHA 0.2 μM	5.66 ± 0.44	1.18 ± 0.13	NA	4.30 ± 0.01	1.03 ± 0.05	NA
SAHA 2 μM	4.67 ± 0.16	1.43 ± 0.09	NA	4.08 ± 0.05	1.08 ± 0.03	NA
Proton SOBP Control	5.37 ± 0.24	NA	1.24 ± 0.07	3.55 ± 0.11	NA	1.25 ± 0.04
SAHA 0.2 μM	4.50 ± 0.01	1.27 ± 0.03	1.26 ± 0.09	3.16 ± 0.13	1.13 ± 0.05	1.38 ± 0.05
SAHA 2 μM	4.16 ± 0.32	1.31 ± 0.05	1.06 ± 0.09	3.15 ± 0.21	1.16 ± 0.09	1.33 ± 0.09
Carbon ion SOBP Control	2.56 ± 0.16	NA	2.59 ± 0.23	2.12 ± 0.04	NA	2.18 ± 0.31
SAHA 0.2 μM	2.24 ± 0.13	1.15 ± 0.16	2.43 ± 0.11	2.17 ± 0.25	1.05 ± 0.20	2.10 ± 0.32
SAHA 2 μM	2.18 ± 0.06	1.18 ± 0.05+	2.12 ± 0.04	2.25 ± 0.08	0.95 ± 0.12	1.82 ± 0.06

NA: Not Applicable.

**Table 2:** Effect of delayed subculture and potentially lethal damage repair with SAHA treatment in A549 and AG1522 cells for different qualities of radiation. D_10_ values are mean ± standard error of the means.

	AG1522, D_10_ (Gy)		A549, D_10_ (Gy)		
SAHA Treatment Started Subculture Time after Irradiation	24 h Before		24 h Before		After Irradiation
	0 h	24 h	0 h	24 h	24 h
γ-rays Control	4.31 ± 0.41	6.35 ± 0.44	6.57 ± 0.37	8.43 ± 0.29	NA
SAHA 0.2 μM	4.49 ± 0.45	6.96 ± 0.36	6.01 ± 0.42	6.97 ± 1.13	8.77 ± 0.41
SAHA 2 μM	4.21 ± 0.73	7.02 ± 0.59	4.85 ± 0.95	6.15 ± 0.59	8.04 ± 0.24
Proton SOBP Control	3.90 ± 0.36	5.35 ± 1.30			
SAHA 0.2 μM	3.78 ± 0.47	5.53 ± 1.90			
SAHA 2 μM	2.81 ± 0.39	4.14 ± 0.89			
Carbon ion SOBP Control	2.11 ± 0.75	3.09 ± 0.13			
SAHA 0.2 μM	2.03 ± 0.25	2.93 ± 0.52			
SAHA 2 μM	1.98 ± 0.06	2.57 ± 0.51			

NA: Not Applicable.

## Abbreviations

SAHA	Suberoylanilide hydroxamic acid
HDACi	Histone deacetylase inhibitor
LET	Linear energy transfer
RBE	Relative biological effectiveness
SER	Sensitization Enhancement Ratio
D_10_	Dose to achieve 10% cell survival
PMRC	Proton Medical Research Center
HIMAC	Heavy ion medical accelerator
SOBP	Spread out Bragg peak
ANOVA	Analysis of valiance
